# Neural oscillations during motor imagery of complex gait: an HdEEG study

**DOI:** 10.1038/s41598-022-07511-x

**Published:** 2022-03-12

**Authors:** Martina Putzolu, Jessica Samogin, Carola Cosentino, Susanna Mezzarobba, Gaia Bonassi, Giovanna Lagravinese, Alessandro Vato, Dante Mantini, Laura Avanzino, Elisa Pelosin

**Affiliations:** 1 Department of Neuroscience, Rehabilitation, Ophthalmology, Genetics, Maternal and Child Health, University of Genoa, 16132 Genoa, Italy; 2 Movement Control and Neuroplasticity Research Group, KU Leuven, 3001 Leuven, Belgium; 3 S.C. Medicina Fisica e Riabilitazione Ospedaliera, ASL4, Azienda Sanitaria Locale Chiavarese, Chiavari, Italy; 4 Ospedale Policlinico San Martino, IRCCS, 16132 Genoa, Italy; 5 National Center for Adaptive Neurotechnologies, Stratton VA Medical Center, Albany, NY USA; 6 Section of Human Physiology, Department of Experimental Medicine (DIMES), University of Genoa, 16132 Genoa, Italy

**Keywords:** Neuroscience, Physiology

## Abstract

The aim of this study was to investigate differences between usual and complex gait motor imagery (MI) task in healthy subjects using high-density electroencephalography (hdEEG) with a MI protocol. We characterized the spatial distribution of α- and β-bands oscillations extracted from hdEEG signals recorded during MI of usual walking (UW) and walking by avoiding an obstacle (Dual-Task, DT). We applied a source localization algorithm to brain regions selected from a large cortical-subcortical network, and then we analyzed α and β bands Event-Related Desynchronizations (ERDs). Nineteen healthy subjects visually imagined walking on a path with (DT) and without (UW) obstacles. Results showed in both gait MI tasks, α- and β-band ERDs in a large cortical-subcortical network encompassing mostly frontal and parietal regions. In most of the regions, we found α- and β-band ERDs in the DT compared with the UW condition. Finally, in the β band, significant correlations emerged between ERDs and scores in imagery ability tests. Overall we detected MI gait-related α- and β-band oscillations in cortical and subcortical areas and significant differences between UW and DT MI conditions. A better understanding of gait neural correlates may lead to a better knowledge of pathophysiology of gait disturbances in neurological diseases.

## Introduction

Gait is no longer considered a simple and automatic motor task, but it requires several non-motor functions (e.g., attention, visuo-spatial abilities). The contribution of these non-motor functions to locomotion is particularly evident in complex walking situations (e.g., avoiding hazards or obstacles or cognitive load that demands attention, planning and dual tasking^1^, where gait must be continuously adapted based on the environmental factors^2^. Beside a precise control over the execution of movement, gait modifications require an appropriate planning of the movement to be made. Indeed, when hazards or obstacles occur, humans have to determine how to modify in advance gait features, such as speed, step length, step height, in order to step over those obstacles while maintaining smooth forward progress and postural stability. Therefore, a correct planning of such precise locomotor movements is crucial for an efficient and safe gait during everyday circumstances and to prevent falls. This is one of the reasons why in the last decade the understanding of neural control of usual and complex gait in humans received considerable attention.

To date, to investigate the neural processes associated with gait control, several imaging (e.g., functional magnetic resonance imaging, fMRI) and neurophysiological techniques (e.g., near-infrared spectroscopy^3^, nuclear neuroimaging^4^ and electroencephalography (EEG)^5^ have been used in combination with motor imagery (MI) paradigms.

Indeed, MI, defined as the mental simulation or rehearsal of an action without its actual execution^6^ is widely used for studying brain activity during walking, given the overlap of neural networks during simulated and actual gait^7–9^. Briefly, MI can be performed via two strategies: (i) visual MI (vMI), during which the subject “sees” movement execution by an internal or an external perspective and (ii) kinesthetic MI (kMI), which implies the feeling of the simulated action^10^.

Regarding usual walking, results from numerous studies^11–16^ are consistent in demonstrating that several cortical (dorsal premotor cortex, superior parietal lobules, posterior rostral cingulate zone) and subcortical (basal ganglia, mesencephalic locomotor region, and cerebellum) regions are activated during gait MI.

In contrast to usual gait, little is known about the cortical contributions of complex gait tasks in healthy subjects. Two studies^17,18^ investigated possible differences in neural activation in healthy subjects during MI of simple (i.e., to imagine walking on a smooth path) and difficult (i.e., to imagine walking on irregular paths) gait tasks with fMRI. Overall, results showed a greater activation of cortical (superior temporal lobules, parietal, and frontal areas) and subcortical areas (right hippocampus and basal ganglia) during complex gait with respect to simple walking task. Later, using functional near-infrared spectroscopy (fNIRS)^19–21^, the specific role of the prefrontal cortex (PFC) during complex gait has been investigated in healthy young and old participants. So far, results are still controversial, however most of the studies reported an increase of PFC activity during complex gait tasks in healthy young subjects and a lower PFC activity in older adults who had difficulties in MI ability.

To our knowledge, no study has yet investigated differences between usual and complex gait motor imagery task in healthy subjects using high-density electroencephalography (hdEEG). In addition to neuroimaging approaches, which have highlighted the structural components of the cortical network engaged during complex walking, EEG can add interesting information about brain oscillations devoted to gait control, thus helping to better understand the functioning of this network. Furthermore, hdEEG has a greater spatial resolution compared to standard EEG and will allow us to gain information on the sources of the electrical oscillations underpinning MI processing with an optimal temporal resolution^22^.

The primary aim of this study was to evaluate the neural correlates of gait MI, looking at the differences in the electrophysiological response, and more specifically in the Event Related Desynchronizations (ERDs), among usual (UW) and dual-task (DT) walking conditions (i.e., obstacle crossing performance) in a population of healthy subjects. Furthermore, we assessed possible relationships between the activation of brain areas during MI tasks and the participants’ imagery ability. Precisely, we focused on analyzing changes in α and β bands, which have been shown to be the predominant oscillations involved in motor preparation, execution or imagination^23^. To this end, we applied a custom developed pipeline for performing source localization from hdEEG data. This pipeline is able to detect multiple brain networks that are spatially similar to those obtained from fMRI data^24–27^.

## Methods

### Subjects

A total of 19 healthy adults (11 females) participated in the study at the Department of Neurosciences (DINOGMI) of the University of Genoa. The age range was 20 to 49 years (mean ± SD: 34.89 ± 12.07). Written informed consent was obtained from all participants prior to the experimental session. Subjects who had experience with MI techniques or MI training were excluded from participation. None of the volunteers had any history of neurological diseases or was being treated with any medication that affected the central nervous system. The study conforms to the standard of the Declaration of Helsinki and was approved by the institutional ethical committee (Comitato Etico Regionle (CER) Liguria Ref.1293 of September 12th, 2018). Demographic characteristics are reported in Table 1.

**Table 1:** Demographic characteristics and behavioural data (mean ± standard deviation).

Demographic characteristics and behavioral data	
Gender (female)	11 (57.89%)
Age (years)	34.89 ± 12.07
Education (years)	19.11 ± 2.19
KVIQ-VI (score)	43.33 ± 6.29
VMIQ-External VI (score)	24.17 ± 9.87
VMIQ-Internal VI (score)	21.89 ± 9.00

*KVIQ* Kinesthetic and Visual Imagery Questionnaire, *VI* Visual Imagery, *VMIQ* Vividness of Movement Imagery Questionnaire-2.

### Experimental design and procedure

#### Baseline assessment

To evaluate MI ability, we used two scales: (i) The Kinesthetic and Visual Imagery Questionnaire (KVIQ)^28^ and (ii) the Vividness of Movement Imagery Questionnaire-2 (VMIQ)^29^. The KVIQ evaluates the subject’s ability to mentally represent movements performed with all body segments. A total of 10 actions are assessed with both the visual and the kinesthetic subscales on a five-point ordinal scale, with a total score range from 0 to 50. The higher the score, the better is the subjective imagery ability. The VMIQ^29^ was designed to assess the vividness of imagery from three perspectives: internal visual imagery, external visual imagery, and kinesthetic imagery^30,31^. We selected this scale because all the 12 imagined actions are related to gait performance or lower-limb movements. The overall score ranges from 12 to 60. The lower the score, the better is the individual’s perceived imagery ability.

#### Motor imagery task

**Figure 1:**
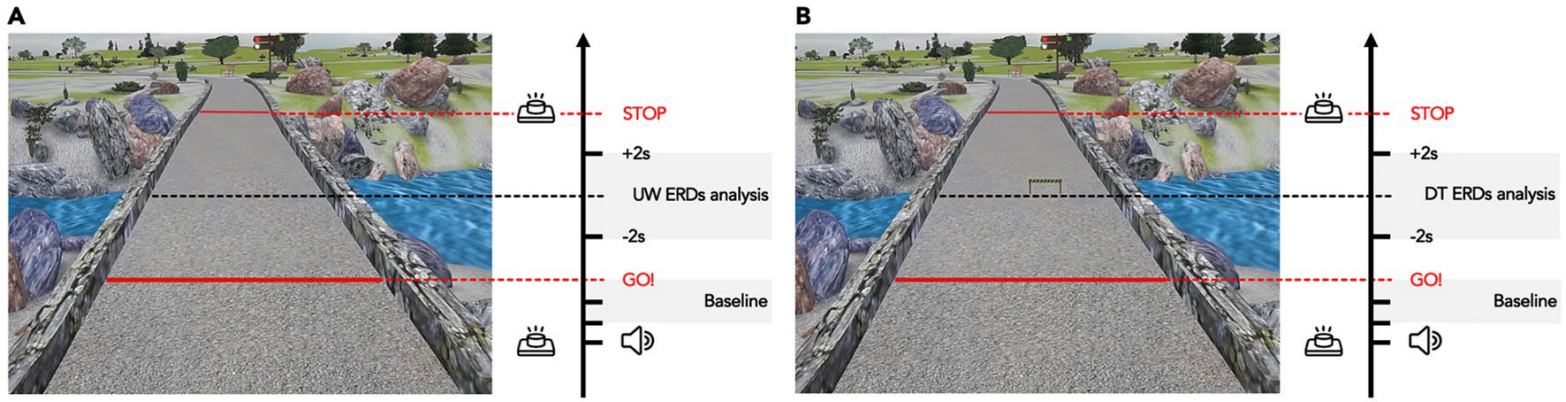
Motor imagery tasks. (**A**) Usual Walking (UW) condition. The picture shows a straight pathway; (**B**) Dual-Task (DT) condition. The picture shows a straight pathway with a hurdle positioned half-way on the right side. The black arrow indicates the task progression. The first red line (GO!) indicates the starting point of MI task; the second red line indicates the end of the MI task (STOP). The grey box (Baseline) indicates the time window selected as ERDs baseline analysis. The two grey boxes (UW and DT ERDs analysis) indicate the time window selected for ERDs analysis during UW and DT conditions.

After the baseline assessment, participants were asked to sit down in front of a computer screen where two pictures representing a straight pathway with no obstacles (Fig. 1A) and a straight pathway with a hurdle positioned half-way on the right side (Fig. 1B) were displayed. In both images two red lines were placed at the beginning and at the end of the walking pathway. Participants were required to perform two MI tasks: (i) to imagine walking on the pathway with no obstacles (Usual Walking; UW); (ii) to imagine walking on the pathway and crossing the hurdle (Dual Task, DT). During both MI tasks, subjects were asked to visually imagine (i.e., vMI) themselves walking at their preferred speed starting from the first red line and stopping at the second red line (Fig. 1A). For the trials in which participants were asked to overcome an obstacle it was specified to visually imagine stepping over the obstacle with their right leg (Fig. 1B). Before each trial, subjects were instructed to press a push-button, start the MI performance at the “GO” of a 4-s audio countdown (“3, 2, 1, GO”) and then press again the button immediately after having passed the second red line. We selected vMI because we were interested in better understanding “higher level processes” involved in gait motor control (such as navigation, planning sequential movements to overcome an obstacle) and because, for this specific task, vMI is the strategy most used in real life situation. A recent paper^32^ investigated the characteristics of kMI and vMI by using connectivity patterns and showed that the primary somatosensory cortex was significantly more activated and centralized in the kMI than in vMI, confirming the specificity of kMI in evoking sensory information of a given movement or action. In contrast, connectivity of premotor cortex was significantly higher in vMI than in kMI, supporting the specificity of vMI in activating areas involved in the planning and preparation of the actual movements. The vMI protocol was performed in a quiet room and participants kept their eyes open during the MI tasks. Each MI task (UW and DT) consisted of 30 trials grouped in 3 blocks (10 trials each) for a total of 60 trials. A 3 s fixation cross was presented on the screen among each trial to avoid mental fatigue. The order of the blocks was randomly assigned, and the duration of the entire experiment was between 25 and 35 min.

### EEG procedures

#### Data recording

Brain activity was recorded using a hdEEG (128-channel) system (Brain Products GmbH, Munich, Germany) with active wet (gel) electrodes set according to the 5–10 system^33^. A trigger box was connected to the system for handling external signals. HdEEG data were recorded at 1000 Hz, using the electrode FCz as physical reference, as recommended by the manufacturer, to better separate and control the contribution and the signal quality of the reference channel. Horizontal and vertical electrooculographic (hEOG/vEOG) signals were also collected to account for ocular-related artifacts in offline EEG analyses. Electrode impedance was checked with the Brainvision Recorder software (Brain Products, https://brainvision.com/products/recorder/) and kept below 5 kΩ throughout the acquisition.

#### Data processing and source localization

HdEEG data were analyzed using an automated pipeline introduced in previous studies^24,26,34,35^ and consisting in three main steps: (i) EEG signal preprocessing, (ii) realistic head model creation, (iii) source reconstruction.

*(i) EEG signal preprocessing* Raw data acquired on the scalp were first processed to identify and correct bad channels, by interpolating their time course from the neighboring channels. Between 0 and 26 channels (median = 7; IQR = 7) mostly located in the frontal part of the EEG cap were corrected in each participant. Then, sensors signals were filtered in the band (1–80 Hz) using EEGLab (https://sccn.ucsd.edu/eeglab). Ocular and muscular artifacts were rejected using the Independent Component Analysis (ICA)^26^. In particular, each IC was classified according to three parameters: the correlation of the power of the IC with the power of vEOG and hEOG signals; the coefficient of determination obtained by fitting the IC power spectrum with a 1/f function; the kurtosis of the IC time-course^24,27^. The thresholds for these parameters were set in accordance with previous studies^24,36^. The time courses of the ICs classified as bad were reconstructed at the channel level and then subtracted from the hdEEG data. The number of artifactual ICs greatly varied among the datasets, in median 51.5 components (IQR = 46) were classified as bad and consequently removed from the channel data. Finally, clean hdEEG recordings were re-referenced using the average re-reference approach^37^.

*(ii) Realistic head model creation.* The leadfield matrix required in the source activity reconstruction step was calculated from a head model built using a template MR head image and template electrode positions^24,25,34^. The MR image was segmented in 12 layers^38^ and the conductivity value of each layer was defined based on previous literature^39^. The matrices describing the transformation between the MNI and the participant’s head space were calculated to account for the individual variability in the EEG positions and allow the statistical comparison between results obtained for different subjects. Template electrode positions were rigidly co-registered to the head contour, defined as the outer layer of the skin compartment. Dipoles corresponding to cortical, subcortical and cerebellar gray matter sources were positioned according to a regular 6 mm grid. Then, the numerical approximation of the whole-head volume conduction model was calculated as a finite element model^40^ by using SimBio (https://www.mrt.uni-jena.de/simbio). Finally, based on this volume conduction model, the leadfield matrix expressing the scalp potentials corresponding to each source configuration was generated.

*(iii) Source reconstruction.* Artifact-free re-referenced scalp hdEEG data and the realistic head-model were provided as input to the exact low-resolution brain electromagnetic tomography algorithm (eLORETA)^41^, in order to estimate brain activity for each voxel within the source space. The eLORETA method is an optimized version of the weighted minimum norm inverse solution, where the weights are unique and the inverse solution provides exact localization for any point source in the brain.

### ERD analysis

For the analysis, we selected 66 regions of interest (ROIs) included in the AAL brain atlas^42^, defined in MNI space, each corresponding to one of the cortical areas that have been most commonly associated with MI of walking^8,9,11,13,15,17,18,43–46^. The list of ROIs corresponding to each mask is reported in the supplementary materials (Table S1). Using the matrix describing the transformation between the MNI and the participant’s head space, calculated in the previous steps, ROI coordinates were transformed into individual space. All voxels included in a spherical region centered in the ROI coordinates, with 6 mm radius, were considered to represent the ROI activity. Specifically, such activity was described by the principal component of these voxels’ time courses and was analyzed in the α (8 ÷ 13 Hz) and the β (13 ÷ 30 Hz) bands separately. The frequency dependent desynchronizations (ERDs) were assessed using source reconstructed data. A time–frequency decomposition was performed on each voxel time-course by means of the Short-Time Fourier Transform, with moving Hamming window of 2 s and 50% overlap between consecutive windows. The resulting spectrogram was generated in the frequency range 1 ÷ 80 Hz, at steps of 1 Hz and epoched with a time window of 4 s.

To run ERD analysis, the mean time required to complete the imagery task, for each participant, was calculated for usual and complex gait separately. Then, a 4-s epoch (+ 2 s; − 2 s) was defined based on the midpoint of each imagery task. This epoch was selected because, prior to the experiment, with a mental chronometry test, we verified that in all participants the obstacle crossing occurred at about half the time needed to complete DT imagery task. Spectrogram epochs of usual and DT MI tasks were then averaged separately. Finally, the ERD intensity was calculated as the percentage value of the relative difference between the epoch power at a given time point and the average baseline power for both α and β bands (Zhao et al.^26^ or $\frac{\mathrm{ERS}}{\mathrm{ERD}\left(\mathrm{f},\mathrm{ t}\right)}=\frac{\mathrm{P}\left(\mathrm{f},\mathrm{ t}\right)-{\mathrm{P}}_{\mathrm{B}}\left(\mathrm{f}\right)}{{\mathrm{P}}_{\mathrm{B}}\left(\mathrm{f}\right)}\times 100\mathrm{\%}$). We chose as baseline the 2000 ms preceding the MI task.

For each condition, ERD spatial maps were created averaging the time–frequency values corresponding to the relevant frequencies within the same range. ERD maps reconstructed in individual space were then converted to MNI space according to the transformation defined on the template MR head image^24,25,34^.

### Statistical analysis

To assess individual ERD intensity changes induced by each MI task, a one-sample t-test between rest and usual walking and rest and dual task MI conditions was performed on the corresponding maps. This analysis was run separately for each ROI and for each frequency band. The significance level *p* was corrected for multiple comparisons according to the FDR procedure^47^ (p_FDR_ < 0.05).

To assess the significant difference between the two MI conditions (UW vs DT) for each participant, frequency band and ROI, we first calculated the number of the desynchronized grey matter voxels [$dsv(i)$] as the number of negative voxels within the i-th ROI mask in the individual desynchronization map. Then, the $dsv(i)$ values were normalized on the total number of voxels within the corresponding mask: $ds\%(i)=100 \cdot \frac{dsv(i)}{\sum vox({mask}_{i})}$^48^. For each ROI, a one-sample one-sided paired t-test was used to test whether the number of desynchronized voxels was higher in the DT condition compared to the UW. All the results were corrected for age. Pearson’s correlation coefficients (R) were used to identify a possible relationship between motor imagery ability (i.e., KVIQ and VMIQ visual imagery scores) and cortical activity changes (i.e., $ds\%(i)$) in DT MI task. Furthermore, a multivariate linear regression analysis was run to evaluate which brain region contributes more to the prediction of MI ability (KVIQ visual subscale, VMIQ-External VI and VMIQ-Internal VI scores). The significance level of the t-test was set to p < 0.05 and to p_FDR_ < 0.05 after correction for multiple comparisons^47^. All analyses were conducted with MATLAB (R2018a, Math-Works, Natick, MA, USA).

## Results

Participants’ demographic, KVIQ and VMIQ sub-scores are summarized in Table 1. The mean education level was 19.11 ± 2.19 years (range 14–21). To specifically measure the visual imagery (VI) ability of each participant we calculated the mean score of the KVIQ visual subscale and of the VMIQ part 1 (Internal VI) and part 2 (External VI) separately. The mean (± SD) sub-scores of the KVIQ visual subscale and of the VMIQ-External VI and VMIQ-Internal VI were 43.33 ± 6.29, 24.17 ± 9.87, and 21.89 ± 9.00 respectively.

### Group-level analysis

All the activations listed below for both the UW and the DT MI conditions resulted to be significant after correction for multiple comparisons (p_FDR_ < 0.05).

#### ERDs during motor imagery of usual and dual task walking with respect to rest

**Figure 2:**
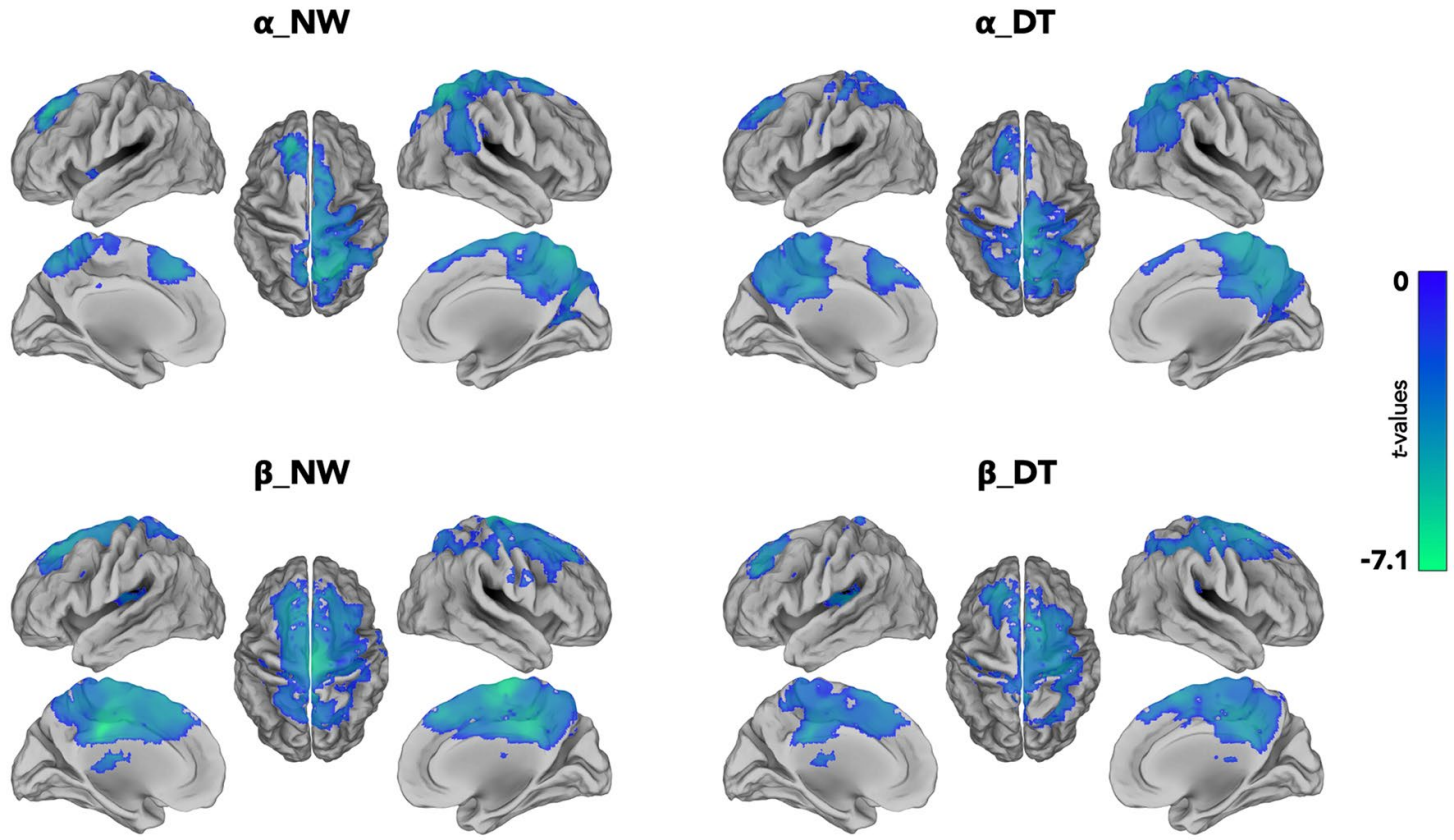
Group-level analysis: significant ERDs (t-values, p_FDR_ < 0.05) in α and β bands in the two imagery conditions.

As shown in Fig. 2 both usual and DT gait MI led to significant activations in several cortical areas involved in MI. The areas that showed significant desynchronization in α and β bands during both gait imagery tasks (usual and dual task gait) with respect to rest are listed below.

- Regarding frontal areas, for usual and DT gait MI both α and β bands showed a significant desynchronization in the superior frontal regions and in the medial frontal regions bilaterally, in the left middle frontal gyrus, in the supplementary motor areas (SMAs), in the right precentral, and in the paracentral lobule bilaterally. Moreover, in β-band only (panels C, D), during both motor imagery tasks, we found a significant desynchronization of the right middle frontal region, and of the left precentral area with respect to rest condition.
- In parietal regions, we found significant α and β bands activation in the right postcentral region, in the right superior parietal lobule, in the right inferior parietal gyrus, in the precuneus bilaterally and in the right angular gyrus for both UW and DT motor imagery tasks respect to rest. For both tasks, α desynchronization in the right supramarginal gyrus and β desynchronization in the left postcentral area were also observed (panels A, B).
- In the temporal and occipital regions, during both MI tasks, significant α and β bands desynchronizations were found in the right superior occipital region whereas in the right middle occipital cortex, in the right cuneus and in the middle part of the right temporal cortex significant ERDs were observed in α-band only.
- Finally, during UW and DT gait MI tasks both α and β bands ERDs were seen in the right middle and posterior cingulate cortices, whereas significant desynchronizations in the left middle and posterior cingulate regions and in the left insular cortex were seen only for β-band.

Below, the areas that showed significant desynchronization in α and β bands specifically during usual gait MI or dual task gait MI with respect to rest.

- DT motor imagery task was characterized by a significant β ERD increase in the supramarginal gyrus bilaterally and in the left superior temporal region (panel D) and by a significant α ERD increase in the left precentral and postcentral areas, in the left superior and inferior parietal regions, in the left cuneus and in the middle and posterior portions of the left cingulate cortex with respect to rest (panel B).
- UW motor imagery task was characterized by β ERD increase in the left superior parietal region, in the left anterior cingulate cortex, and, subcortically, in the left caudate and by α ERD increase in the right superior temporal cortex with respect to the rest condition (panel C).

### Subject-level analysis

#### Usual walking vs dual task imagery conditions

The comparison of the % of desynchronized grey matter voxels (i.e., *ds%*) between the two gait imagery tasks was performed for cortical areas showing significant desynchronization in α and β bands (Table 2). We found that several areas of the motor imagery network were more largely desynchronized, in both α and β-bands during imagination of DT gait respect to usual walking. Particularly, statistical analysis revealed a larger β-band desynchronization during DT gait compared to usual gait imagery tasks in the following brain regions: right precentral (*p* = 0.048), right superior and inferior parietal gyri (*p* = 0.04 and *p* = 0.01 respectively), and bilateral precuneus (left *p* = 0.04; right *p* = 0.02). Furthermore, increased α-band activity in the left postcentral gyrus (*p* = 0.01) and in the left inferior parietal area (*p* = 0.04) were found during DT gait task with respect to usual walking condition. Finally, no area showed a higher percentage of desynchronized grey matter voxels during usual walking with respect to DT gait imagery tasks. The resulted reported above did not remain significant after FDR correction.

**Table 2:** Differences in ds% in the two imagery conditions.

Mask	x	y	z	*ds%* UW	*ds%* DT	*p*_*unc*_	*p*_*FDR*_	*t-value*
**Beta band**								
Precentral R	41	− 8	52	59.13 (14.69)	63.90 (15.79)	0.048	0.38	− 1.75
Parietal Sup R	26	− 59	62	49.59 (19.12)	57.20 (9.58)	0.04	0.38	− 1.89
Parietal Inf R	46	− 46	50	66.72 (25.46)	83.86 (6.42)	0.01	0.31	− 2.79
Precuneus L	− 7	− 56	48	65.62 (23.35)	74.11 (15.05)	0.04	0.38	− 1.80
Precuneus R	10	− 56	44	59.52 (22.93)	69.75 (10.39)	0.02	0.38	− 2.27
**Alpha band**								
Postcentral L	− 42	− 23	49	50.23 (27.78)	63.68 (21.30)	0.01	0.47	− 2.38
Parietal Inf L	− 43	− 46	47	56.18 (31.02)	69.39 (22.41)	0.04	0.57	− 1.83

Reported p values refer to DT > UW comparison.

ds% = normalized % of desynchronized voxels within the corresponding mask, UW = Usual Waking imagery, DT = Dual Task imagery, p_unc_ = uncorrected p values, p_FDR_ = FDR corrected p values, L = left, R = right.

### Correlations

#### MI ability tests and ds%(i)

**Figure 3:**
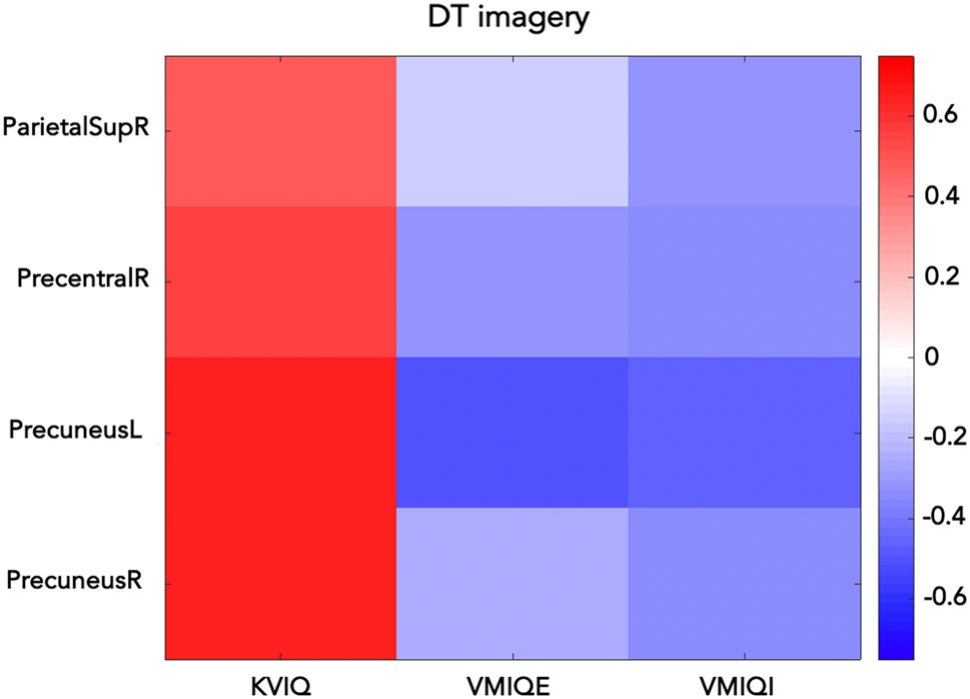
β band correlations between MI ability test scores and ds%.

Correlations results are shown in Fig. 3. When the percentage of desynchronized grey matter voxels (i.e., *ds%(i)*) was correlated with the imagery ability scores, significant correlations during complex gait MI task (DT) in the β frequency band were observed. Specifically, we found significant correlations between KVIQ score and *ds%* of the right precentral (r = 0.550, *p* = 0.018) and superior parietal areas (r = 0.480, *p* = 0.044), and of the bilateral precuneus (left: r = 0.65, *p* = 0.004; right: r = 0.635, *p* = 0.005). The multivariate linear regression analysis revealed that the *ds%* of the left precuneus was the only independent variable to explain KVIQ score (Beta 0.65, p = 0.004, IC 0.247–1.051). Finally, a negative significant correlation between left precuneus *ds%* and the VMIQ-E score (r = − 0.494, *p* = 0.037) was also found.

## Discussion

The purpose of this study was to better investigate the neural substrate of complex walking using a MI paradigm in a sample of healthy adults’ population. To this aim, we compared temporal dynamics and spatial location of α-band and β-band neural oscillations associated to usual and dual-task visual gait motor imagery measured with hdEEG.

In line with previous studies^11–21^, during usual gait MI task, we found significant activation of several frontal, parietal, temporal and occipital areas, and cingulate cortex. As the task became more difficult (i.e., dual-task MI condition) an increased activity of specific brain regions in both α and β bands was also observed. Finally, exploring possible relationships between motor imagery perceived ability and changes in neural activation, the percentage of desynchronized grey matter voxels in β-band in the left precuneus was the only independent variable to explain KVIQ score whereas the percentage of desynchronized grey matter voxels in β-band in the left precuneus correlated with VMIQ-E score.

First, both gait imagery tasks activated a large cortical-subcortical network encompassing mostly frontal, parietal, temporal, occipital, and cingulate cortex. Indeed, for both tasks, we found ERDs both in α and β bands in the superior and medial frontal regions bilaterally, left middle frontal gyrus, SMAs, right precentral and postcentral areas and paracentral lobules, bilateral precuneus, right superior and inferior parietal areas, the right angular gyrus, occipital areas and the right cingulate cortex compared with the rest condition. Furthermore, during both MI task execution, we observed an increased activation in β-band in the right middle frontal gyrus and in the left precentral, in the left postcentral area, in the left middle and posterior cingulate regions and in the left insular cortex and an increased ERD in α -band in the right middle occipital, right cuneus, and right middle temporal regions.

Concomitant α and β desynchronizations in sensorimotor areas have been consistently reported in previous studies^49–51^. Alpha oscillations are classically considered to reflect an “idling” cortical state that is interrupted by motor or sensory processes^52,53^, resulting in alpha ERD. However, it has been also suggested that α may index the active inhibition of sensory information during internally directed attentional tasks such as mental imagery^54^. For example, in order to facilitate mental imagery, one needs to inhibit or ‘reject’ incoming sensory information. Ray and Cole^55,56^ found increased α power in rejection tasks such as mental imagery and arithmetic especially at parietal sites. ERDs in α band only, observed for UW and DT gait imagery task in a number of parietal, temporal and occipital regions can be interpreted either as reflecting possible interruption of the resting state and as active inhibition of sensory information and multimodal sensory processing.

Beta ERD is seen in motor planning around movement onset, both for motor execution and imagination^57–59^. β ERD may indicate that a large part of the sensorimotor network is engaged in both the imagery tasks. Concerning MI tasks, the activation of frontal areas (such as SMA) has been related to the mental representation of motor action per se^60^. Furthermore, it has also been shown that during mental simulation of walking, the activation of SMA and pre-SMA was specifically associated with gait initiation and with the maintenance of proper sequencing and timing of limb movements^61^. The parietal cortex, particularly the precuneus, was shown to be involved with visuo-spatial processing and attention processes, strongly required during mental simulation of walking. Similarly, increased activity in occipital areas during gait MI tasks was associated with visuo-spatial navigation and imagination of visual environment^62–64^.

Moreover, our results demonstrated that other areas showed significant ERD in α and β bands specifically during UW or DT gait imagery with respect to rest and that some cortical areas were more largely desynchronized in DT respect to UW MI task.

For UW motor imagery task, we found β ERD in areas involved in aspects of attention and visuo-spatial perception as the left superior parietal and left anterior cingulate cortex and, subcortically, in the left caudate and α ERD in the right superior temporal cortex. Several studies, using fMRI technique, supported the involvement of the basal ganglia during motor imagery tasks, particularly for automatic or repetitive movements^65–68^. In this regard, the activation of the basal ganglia-thalamo-motor cortical circuit was constantly reported during the imagination of human gait^9,17^, corroborating the idea that acquired automatic movements rely on the activation of this network.

For DT vMI task, we found significant ERD in β band in the supramarginal gyri and in the left superior temporal region and a significant α ERD increase in the left precentral and postcentral areas, in the left superior and inferior parietal regions, in the left cuneus and in the middle and posterior portions of the left cingulate cortex with respect to rest.

When cortical activity during usual and complex vMI task were compared, we observed a larger ERD, in terms of number of desynchronized voxels, in β band in the right precentral area, right superior and inferior parietal gyri, bilateral precuneus and larger ERD in α band in the left postcentral gyrus and in the left inferior parietal areas with respect to UW. No area showed a higher percentage of desynchronized grey matter voxels during UW with respect to DT gait imagery task.

Regarding the larger frontal β-band activations during DT MI task respect to UW, our results are in line with previous findings exploring differences on neural correlates during usual and complex gait tasks. fNIRS studies revealed increased activity^69^ in these areas when subjects had to perform challenging walking compared to simple gait. Furthermore, a greater activation of prefrontal areas was also found during a complex gait imagery task. Van der Meulen and colleagues^17^, using fMRI data, showed a higher prefrontal activity when subjects were required to imagine walking on an irregularly surfaced path compared to a smooth one. Taken together, these findings support the idea that as the task became more difficult, the frontal areas became essential in the higher-order cognitive control of gait^17^. For the larger β-band ERD observed in the parietal cortex and the larger ERD in α band in left postcentral area and in right precuneus in DT with respect to UW, evidence strongly points to the role of the parietal cortex during motor imagery^70^, not only for sensory information processing and visuospatial navigation^65,71–73^ (when visual information is required) but also for coding actions goals^74,75^ and movements preparation, redirection, and intention^76^. Hence, the larger ERD in β-band within the parietal cortex observed during DT gait MI could likely reflect the higher cognitive demands required for accomplishing a more complex task^77^. Indeed, a greater activation of the precuneus was already observed in previous studies investigating changes in cortical activity induced by increasing the complexity of the imagery tasks^14,15,45,78^ as well as during the imagery of complex gait, such as walking in presence of obstacles^13,79^.

A further result was that in most of the areas that showed a larger ERD, in terms of number of desynchronized voxels (*ds%*), in β band in DT respect to UW (right precentral, right parietal superior and bilateral precuneus) we found significant correlations between *ds%* during DT imagery task and KVIQ and VMIQ scores, showing that the higher was the volunteer’s perceived ability in performing MI, the larger were the desynchronized areas. However, the results of multivariate linear regression analysis showed that the *ds%* of the left precuneus was the only independent variable to explain KVIQ score. Notably, activity in the left precuneus also correlated with VMIQ-E score, supporting the crucial role exerted by this area in the imagery of complex gait.

Specific activations in the β band were observed for the DT gait imagery task only in the supramarginal gyri and in the left superior temporal region with respect to rest. These areas have been shown to be involved in allocentric processing, that is crucial when the obstacle overcoming should be planned^80^. Finally, we found specific ERD in α-band in parietal and cingulate cortex during DT gait imagery task. α ERD has been associated to tasks requiring processing of relevant information in a variety of cognitive domains, but especially linked with visuo-spatial processing. It was thus hypothesized that the suppression of alpha activity may be related to the strength of attention to external objects or stimuli required by the task^81^. However, it is noteworthy to mention on the potential impact of age heterogeneity of our study group (age range: 20 to 49 years) on the results. Indeed, it has been reported that MI ability might change with age, reflecting functional changes in the aging brain^82^. Although all the above reported results were corrected for age, it would be interesting in future studies to investigate ERD/ERS changes in MI-related brain areas across lifespan.

In this study there are some limitations that need to be considered. First, the limited sample size and the age heterogeneity decrease the strength of our results. Second, here, in line with previous studies^24,26,34^ we performed source localization using the eLORETA algorithm, however it should be considered that each source localization method has different effects on EEG connectivity estimates^83^. Third, although cerebellar activity has often been reported in relation with neural correlates of MI, we did not find significant activation neither in α nor in β bands during the whole MI task. Although we used a high-density EEG montage, the ability of hdEEG and MEG techniques in detecting signals from the cerebellum is still under question^84^. Forth, due to the limited sample size the results related to the subject-level analysis, performed to compare usual vs complex gait imagery conditions, did not survive the correction for multiple comparisons. Nevertheless, our results indicate a trend of desynchronizations that, based on the available literature is coherent with the type of MI we analyzed. In order to compensate for the individual variability in the execution of the imagery, more participants will be needed to guarantee enough statistical power to confirm our findings also after correction for multiple comparisons. Alternatively, the inter-subject variability could be accounted for by using frequency bands defined from the individual alpha peaks. Although this strategy does not permit to compare the band-specific desynchronization patterns at group level, it is an interesting approach to investigate how the MI-induced desynchronizations vary with the difficulty of the task within the same participant^84^. Fourth, our set-up lacked of leg muscle activity recording during EEG signal acquisition. However, we might assume that leg muscle activity during MI task had a negligible influence on EEG data since it has been recently demonstrated that EMG activity of distal leg muscles decreased during gait imagery tasks in the sitting whereas standing gait imagery tasks had faciliatory effect on proximal lower limb muscle activity^85^.

Our study provided new insights on the cortical contributions of usual and complex gait in healthy subjects by means of a MI paradigm. Overall, the results showed an increased activation of frontal, parietal and temporo-occipital areas when subjects imagined a challenging walking task compared to a simple gait, supporting the idea that complex gait requires several non-motor functions (e.g., attention, visuo-spatial abilities). Furthermore, to the primary aim of the study, here we analyzed alpha and beta frequency band only, but new mechanism associated with gait MI may be described by studying other frequency bands. However, a better understanding of neural correlates underlying gait performance in healthy subjects may lead to a better knowledge of the pathophysiological mechanism of gait disturbances in patients with neurological dysfunctions.

## Supplementary Information

Supplementary Table 1.

## Data Availability

Data supporting these findings are available from the corresponding author upon reasonable request.
