# Supplementary material for: Neural oscillations during motor imagery of complex gait: an HdEEG study
Source: Sci Rep. 2022 Mar 12;12:4314. doi: 10.1038/s41598-022-07511-x (PMC8918338; doi:10.1038/s41598-022-07511-x)
Supplement: Supplementary file 1 — Supplementary Table 1. [file 41598_2022_7511_MOESM1_ESM.pdf]

Table 1s. Masks selected from AAL atlas, regions they belong, and corresponding MNI coordinates

Brain region	Cluster	Area	Side	X	Y	Z
Frontal area	Superior Frontal area	Superior Frontal Gyrus	L	−18	35	42
			R	22	31	44
		SFG-Orbital part	L	−17	47	−13
			R	18	48	−14
	Middle Frontal gyrus	Middle Frontal Gyrus	L	−33	33	35
			R	38	33	34
		MFG-Orbital part	L	−31	50	−10
			R	33	53	−11
	Inferior Frontal gyrus	Opercular part	L	−48	13	19
			R	50	15	21
		Triangular part	L	−46	30	14
			R	50	30	14
		Orbital part	L	−36	31	−12
			R	41	32	−12
	Medial Frontal gyrus	Superior Medial Frontal gyrus	L	−5	49	31
			R	9	51	30
		Orbital	L	−5	54	−7
			R	8	52	−7
	Supplementary Motor Area		L	−5	5	61
			R	9	0	62
	Precentral gyrus		L	−39	−6	51
			R	41	−8	52
Parietal area	Postcentral gyrus		L	−42	−23	49
			R	41	−25	53
	Superior Parietal gyrus		L	−23	−60	59
			R	26	−59	62
	Inferior Parietal gyrus		L	−43	−46	47
			R	46	−46	50
	Supramarginal gyrus		L	−56	−34	30
			R	58	−32	34
	Precuneus		L	−7	−56	48
			R	10	−56	44
Temporo-Occipital areas	Superior Temporal gyrus		L	−53	−21	7
			R	58	−22	7
	Middle Occipital gyrus		L	−32	−81	16
			R	37	−80	19
Cingulate cortex	Cingulate gyrus	Anterior	L	−4	35	14
			R	8	37	16
		Middle	L	−5	−15	42
			R	8	−9	40
		Post	L	−5	−43	25
			R	7	−42	22
Insula	Insula		L	−35	7	3
			R	39	6	2
Basal ganglia	Caudate		L	−11	11	9
			R	15	12	9
	Putamen		L	−24	4	2
			R	28	5	2
Cerebellum	Cerebellum	III	L	−8	−37	−19
			R	13	−34	−19
		IV-V	L	−14	−43	−17
			R	18	−43	−18
		VI	L	−22	−59	−22
			R	26	−58	−24
		VIIb	L	−31	−60	−45
			R	34	−63	−48
		VIII	L	−25	−55	−48
			R	26	−56	−49
		IX	L	−10	−49	−46
			R	10	−49	−46
		X	L	−22	−34	−42
			R	27	−34	−41
	Cerebellar Crus	Crus 1	L	−35	−67	−29
			R	38	−67	−30
		Crus 2	L	−28	−73	−38
			R	33	−69	−40

L = left, R = right, SFG = Superior Frontal Gyrus, MFG = Middle Frontal Gyrus
